# Influence of different factors on coseismic deformation of the 2015 Mw7.8 earthquake in Nepal

**DOI:** 10.1038/s41598-024-60084-9

**Published:** 2024-04-26

**Authors:** Rui Wu, Xibin Dong, Bo Xia, Weisi Wang, Xiayu She, ZiMing Chu

**Affiliations:** 1 South Surveying and Mapping Technology CO., LTD, Guangzhou, 510000 Guangdong China; 2 Shenzhen International Graduate School, Tsinghua University, Shenzhen, 518000 Guangdong China; 3 College of Geomatics, Xi’an University of Science and Technology, Xi’an, 710054 Shaanxi China; 4 Henan Earthquake Agency, Zhengzhou, 450018 Henan China

**Keywords:** Spectral-element method, Numerical simulation, Coseismic deformation, Coseismic slip distribution, Topographic effect, Seismology, Geophysics, Geodynamics

## Abstract

In Geophysics, topographic factors are observations that can be directly measured, but they are often ignored to simplify the model. Studying the coseismic deformation caused by earthquakes helps accurately determine the epicenter's parameterization. It provides a reference for the reasonable layout of coseismic observation stations and GNSS observation stations. After the Mw7.8 earthquake in Nepal in 2015, GCMT, USGS, GFZ, CPPT, and other institutions released their epicenter parameter. However, according to their parameters, the coseismic displacements simulated by the spectral-element method are quite different from the GNSS observations. Firstly, this paper inverts the geometric parameters of the seismogenic fault with Nepal’s coseismic GNSS displacement. The spectral-element method determines the source's location and depth under the heterogeneous terrain and outputs the source parameters. Among the results of many studies, the surface source is more consistent with the generation mechanism of large earthquakes. Secondly, this paper calculates the fault slip distribution of this earthquake using SDM (Steepest Descent Method) based on GNSS and InSAR data, which is divided into 1500 subfaults, and the moment tensor of each subfault is calculated. This paper investigates the distribution characteristics of the coseismic deformation field of the 2015 Mw 7.8 earthquake in Nepal under three different models. The results show that the influence of topographic factors is ~ 20%, and the influence of heterogeneous factors is ~ 10%. This paper concludes that the influence of topographic factors is much more significant than that of heterogeneous factors, and the influence of both should be addressed in coseismic deformation calculations.

On April 25, 2015, an earthquake of Mw 7.8 occurred near Kathmandu, the capital of Nepal, which brought great disasters to the surrounding countries. The death toll exceeded 8786 and attracted much social attention. Inversion of geometric parameters and slip distribution of seismic faults based on surface deformation information is crucial for exploring earthquake occurrence and preparation mechanisms^1^. After the Mw 7.8 earthquake in Nepal, many scholars provided various coseismic slip fault models based on InSAR or GNSS data^2–6^, which provided many reference data for this earthquake. To simplify the relationship model between fault movement and surface deformation, ignoring terrain factors will bring more significant uncertainty to the inverted fault model.

The practical methods to study terrain effects include the acceptance height function method and numerical simulation methods. The acceptance height function method modifies the fault depth of the homogeneous elastic half-space to ensure that the depth from the calculation point to the fault center is correct. This method can solve the terrain effect problem when the undulation is slight. However, its results could be more reliable when the terrain gradient is significant^7^. In recent years, the spectral element method combines the finite element method, pseudo-spectral method, and GLL (Gauss–Lobatto-Legendre), which has been used to study the propagation of simulated seismic waves^8,9^. Komatitsch has used the spectral element method to analyze the influence of topography and basin structure of the Los Angeles basin on strong ground motion characteristics from the spatial distribution of maximum surface displacement, velocity, and acceleration^10^.

Scholars have done meaningful work on the influence of topographic factors on coseismic deformation. Some scholars believe that topographic factors significantly impact coseismic deformation. For example, the topography of the eastern edge of the Qinghai Tibet Plateau had a maximum effect of 9% on the 2008 Wenchuan Mw7.9 earthquake^11^, and the Japanese trench and seamount had an effect of more than 30% on the 2011 Mw9.0 earthquake in Japan. The local topography even reaches 90%^12,13^. Other scholars believe that topographic factors have a negligible impact on the coseismic deformation of earthquakes, at least much less than heterogeneous factors. For example, the 2009 Mw 6.3 L'Aquila earthquake^14^ and the slow sliding event at the subduction edge of Hikurangi, New Zealand^15^. Langer studied the coseismic deformation using the spectral element method based on the focal mechanism solution, and the results proved that topographic factors could change the distribution pattern of the coseismic deformation field^16^. Li and Barnhart explored the maximum influence of topographic factors and crustal heterogeneity factors of 4% and 8%. They believed that the former affects the distribution form and range of the displacement field^17^. The latter affects the magnitude of displacement. Tung and Masterlark found that the data residuals in the heterogeneous model were smaller and more uniform than those in the homogeneous model^18,19^, which better matched the deformation in the geological complex domain. The southern part of Nepal is an alluvial plain. The other three sides are surrounded by mountains, with a maximum height difference of more than 4000 meters^20^ (Fig. 1). Many earthquake damage surveys, vital earthquake observations, and theoretical studies show that the topographic relief caused by the basin mountain structure significantly impacts the coseismic displacement simulation^21^. Therefore, it is significant to study the influence of topographic factors on the coseismic deformation of this earthquake. Based on previous research results, this paper first determines the optimal source parameters using the coseismic GNSS observation and DEM data of the Nepal Mw7.8 earthquake. Then, it inverts the coseismic slip distribution and delineates subfaults. Finally, it discusses the impact of terrain factors on this earthquake, and a simple risk analysis is conducted on the range affected by the earthquake, hoping to provide a reference for the layout and observation period of earthquake observation stations.

**Figure 1:**
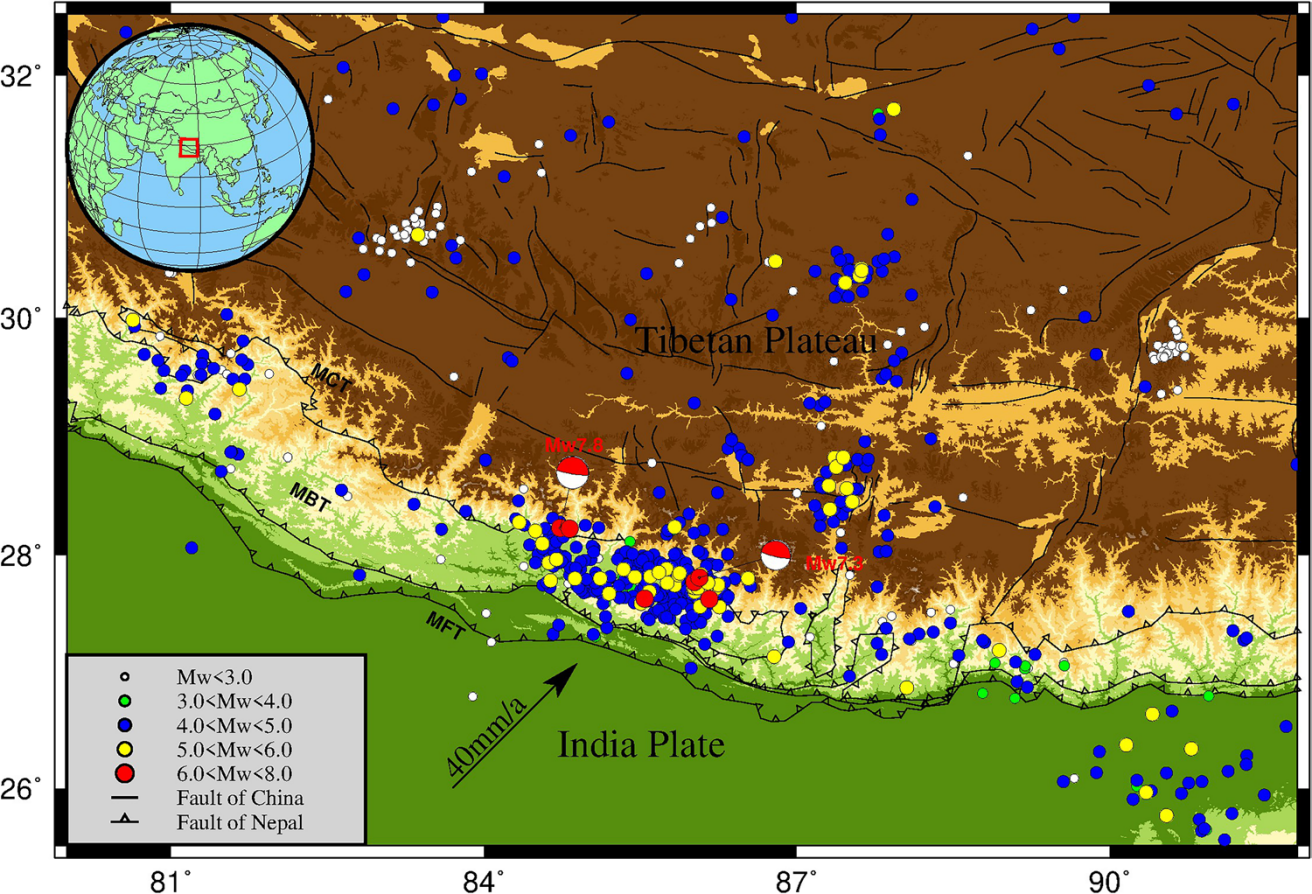
Nepal study area. The thin black lines with triangles in the figure are MFT (main front thrust fault), MBT (main boundary thrust fault), and MCT (main central thrust fault) from south to north. The rest of the thin black lines are fault zones in China. This paper collects 1947 seismogenic solutions for the area around Nepal from January 1, 1980, to September 1, 2022, on the official website of the USGS (https://earthquake.usgs.gov/ ). Solid circles of different colors represent earthquakes of different magnitudes. The two red seismic mechanism spheres represent the earthquake and its most significant aftershocks.

## Data

### Geodetic observation data and topographic data

This research collected 46 GNSS coseismic displacement data, including 13 stations in Nepal and 33 stations in China. According to 13 coseismic displacement data in Nepal by Galetzka et al.^4^, the maximum horizontal error is 6 mm, and the maximum vertical error is 11 mm. The GNSS horizontal coseismic displacement moves southward, and the North–South component is greater than the East–West component. The largest displacement station, KKN4, moves 1.83 m southward and 0.4 m westward; NAST, CHLM, and KIRT stations near the epicenter move 1.3 ~ 1.47 m to the south and 0.2 ~ 0.34 m to the West. According to 33 GNSS coseismic displacement data in China by Wu et al.^22^, the maximum horizontal error is 2 mm, and the maximum vertical error is 7.8 mm.

We use InSAR data from the ALOS-2 T048 track processed by Lindesy using GMTSAR^23^, with the maximum uplift exceeding 1.2 m and the maximum subsidence exceeding 0.7 m. Because the data volume of InSAR is too large for subsequent calculations, we use gradient-based quadtree downsampling to process the InSAR data and reduce the deformation. Data volume while presenting the deformation characteristics of this earthquake.

The topographical grid file uses ETOP1 data from NOAA (the National Oceanic and Atmospheric Administration), a global comprehensive water depth terrain digital elevation model^24^.

### Parameters of vertically layered media

This paper uses the multi-layer elastic half-space earth model in the Himalayan region to construct the grid model. The model is divided into two types of isotropic elastic bedrock materials along the depth (Table 1). An empirical formula determines its wave velocity model (P wave velocity $v_{p}$, S wave velocity $v_{s}$, and quality factor $Q_{\mu }$)^25^.

1 $$\begin{array}{*{20}c} {v_{s} = v_{p} \left( {1.732\sim 2} \right)} \\ \end{array}$$

2 $$\begin{array}{*{20}c} {Q_{\mu } = 0.05v_{s} } \\ \end{array}$$

3 $$\begin{array}{*{20}c} {Q_{p}^{ - 1} = \left( {1 - \frac{{v_{s}^{2} }}{{v_{p}^{2} }}} \right)Q_{k}^{ - 1} + \left( {\frac{{v_{s}^{2} }}{{v_{p}^{2} }}} \right)Q_{\mu }^{ - 1} } \\ \end{array}$$

4 $$\begin{array}{*{20}c} {Q_{s}^{ - 1} = Q_{\mu }^{ - 1} } \\ \end{array}$$

**Table 1:** Model medium parameters.

Material-id	Deep (km)	$\rho$(kg/m^3^)	${v}_{p}$(m/s)	${v}_{s}$(m/s)	${Q\_}_{kappa}$	${Q}_{\mu }$
1	0 ~ surface	2530	5500	3200	655	160
2	− 6 ~ 0	2640	5850	3400	694	170
3	− 12 ~ − 6	2690	6000	3500	723	175
4	− 18 ~ − 12	2830	6450	3700	726	185
5	− 36 ~ − 18	2900	6650	3850	776	192.5
6	− 50 ~ − 36	3070	7200	4150	826	207.5

## Method

### Bayesian inversion of fault geometry

As we all know, when using the spectral element method, the input data are solution parameters of the source mechanism of the earthquake. Traditional inversion methods only get the best single-point estimation model. It is difficult to comprehensively invert the model information in the data, and the obtained inversion results are still being determined. Bayesian inversion provides a natural framework for solving such problems and have the advantage of dealing with multi-dimensional parameters and their uncertainties, so it is widely used in the study of crustal deformation and fault movement^26,27^. The random variables are observation data and model parameters. The posterior probability density function can contain all the prior information of the existing model. The inversion problem is transformed into the information extraction of the posterior probability distribution through Bayesian criteria.

5 $$\begin{array}{*{20}c} {P\left( {m{|}d} \right) = \frac{{{\text{p}}\left( {d{|}m} \right){\text{p}}\left( m \right)}}{{{\text{p}}\left( d \right)}}} \\ \end{array}$$

where d is the observed value column vector; M is fault slip; ${\text{p}}\left( {d{|}m} \right)$ is the likelihood function of d after m is given, and its practical significance is the residual between the observed data and the simulation calculation value; ${\text{p}}\left(m\right)$ represents prior information of model parameters; ${\text{p}}\left(d\right)$ is a constant independent of m.

When the data error $\in$ has a multivariate Gaussian distribution and has zero means, the covariance matrix ${\Sigma }_{d}$ satisfies $\epsilon \sim N\left( {0,\Sigma_{d} } \right)$, and the likelihood function ${\text{p}}\left( {d{|}m} \right)$ is expressed as:

6 $$\begin{array}{*{20}c} {p\left( {d{|}m} \right) = \left( {2{\uppi }} \right)^{{ - \frac{{\text{N}}}{2}}} \left| {{\Sigma }_{d} } \right|^{{ - \frac{1}{2}}} \times exp\left[ { - \frac{1}{2}\left( {d - Gm} \right)^{{\text{T}}} {\Sigma }_{d}^{ - 1} \left( {d - Gm} \right)} \right]} \\ \end{array}$$

where ${\text{N}}$ represents the number of data points, ${\Sigma }_{d}^{ - 1}$ represents the number of data points, and the inverse matrix of the observed data variance–covariance.

### Calculation of coseismic deformation based on the Spectral Element Method

The spectral element method effectively integrates the pseudo-spectral and finite element methods. In a simulated earthquake, its displacement field u satisfies the following fluctuation equation and its weak form in space G multiplied by an arbitrary test function ω^28^:

7 $$\begin{array}{*{20}c} {\sigma = C:\nabla u} \\ \end{array}$$

8 $$\begin{array}{*{20}c} {\rho \ddot{u} = \nabla \cdot\sigma + f} \\ \end{array}$$

9 $$\begin{array}{*{20}c} {\mathop \smallint \limits_{G} \rho \omega \cdot u d^{3} x = - \mathop \smallint \limits_{G} \nabla \omega :\sigma d^{3} x + \mathop \smallint \limits_{G} \omega \cdot fd^{3} x} \\ \end{array}$$

where $\sigma$ is the stress tensor, $C$ is the fourth-order elastic tensor, $\nabla$ is the spatial gradient operator, $u$ is the displacement vector of the mass, $\rho$ is the density, $\ddot{u}$ is the second derivative of $u$, $f$ is the source term, is the two-point multiplication in the tensor operation, and $\cdot$ is the operator of the tensor dot product.

In order to obtain the integration results of Eq. (10), firstly, the study area is divided into several hexahedral cells. Secondly, the GLL product rule is introduced to each hexahedral cell so that each computational cell's mass matrix and stiffness matrix can be formed. Finally, all computational cells are integrated into a whole, and then the results of the numerical simulation of the wave field are obtained:

10 $$\begin{array}{*{20}c} {\user2{M\ddot{u}}({\varvec{t}}) + {\varvec{K}} {\varvec{u}}({\varvec{t}}) = {\varvec{F}}} \\ \end{array}$$

${\mathbf{M}}$ is the global quality matrix; $\user2{\ddot{u}}({\varvec{t}})$ is the global displacement vector; ${\mathbf{K}}$ is the worldwide stiffness matrix; ${\mathbf{F}}$ is the source term.

## Result

Using the natural terrain and three-dimensional elastic structure, we also use a numerical model to estimate the coseismic surface deformation caused by the 2015 Nepal earthquake. Based on the homogeneity of the study area in the longitudinal layered medium, we analyzed the vertical displacement of the surface in the study area.

### Mechanistic solutions for different seismic sources

**Figure 2:**
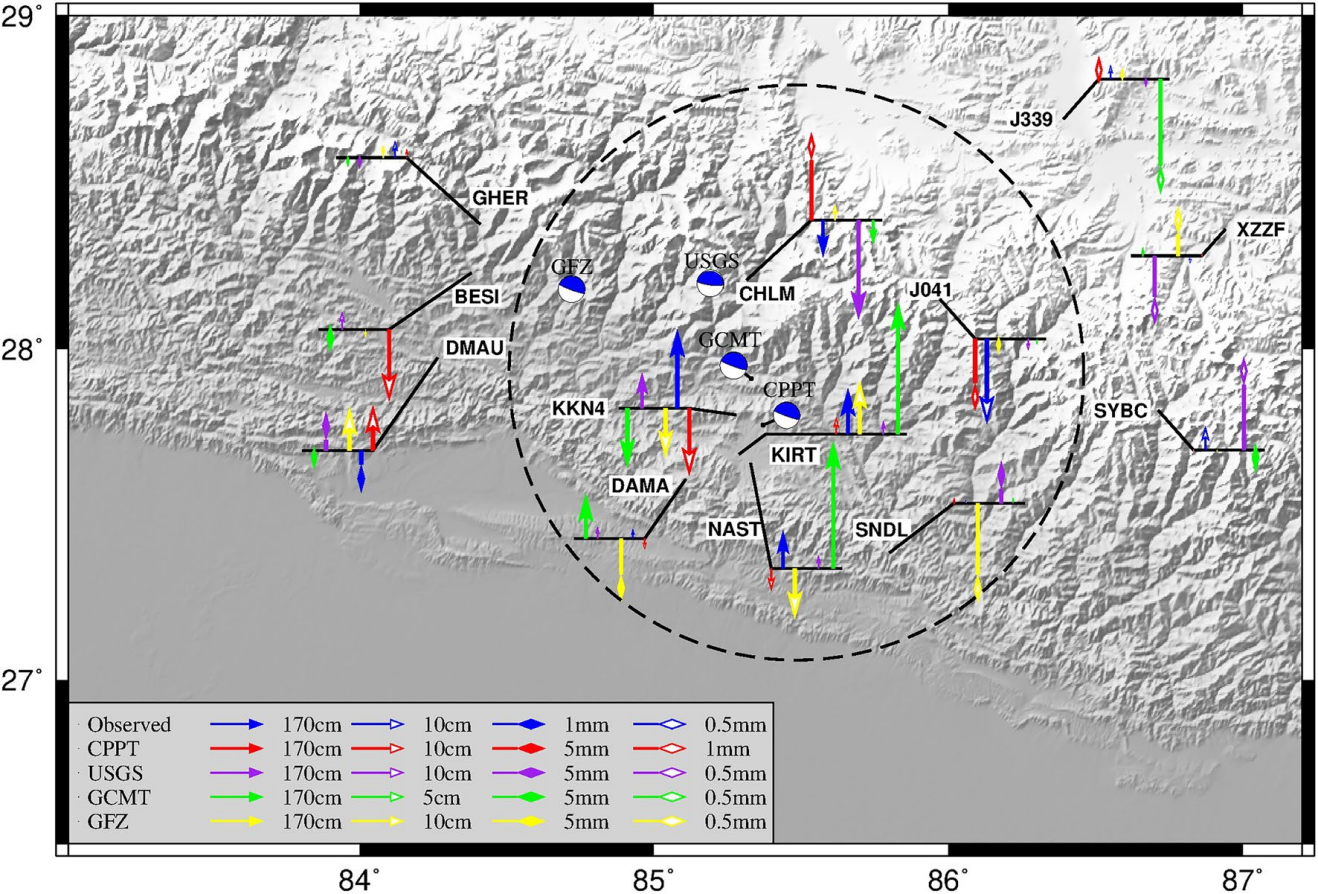
Surface vertical displacement forward based on different focal mechanism solutions. In the figure, the beachball represents the source mechanism solution, the blue color represents the actual GNSS observations, and the red, purple, green, and yellow colors represent the GNSS point displacements corrected using the source mechanism solution of different agencies, respectively. The black dashed circles are the areas with larger surface displacements.

In Fig. 2, the vertical displacements of the sites calculated based on each agency's (in Table 2) seismic mechanism solution differ from the observed values. The focal mechanisms given by different institutions are all thrust rupture earthquakes^29^. For the stations within the black dotted circle, the results from other agencies, except for the USGS results, do not agree with the observed values in the direction. For example, at KKN4, KIRT, NAST, and DAMA, the USGS results and the actual observed values are all in the upward direction, while the results of GFZ, CPPT, and GCMT are all in the downward direction. However, the vertical displacements of the CHLM calculated based on the USGS are more than twice as large as the actual observed values, and the difference in KKN4 is even more significant. The displacements of each site calculated based on the seismic mechanism solution in this paper are close to the same direction as the actual observed values for the stations outside the black dotted line, except for GHER and J339. This may be due to the significant difference in lateral inhomogeneity between the southern and northern edges of the Tibetan Plateau.

**Table 2:** Focal mechanism of the 2015 Nepal Mw7.8 earthquake.

Source ^30–32^	Lon(°)	Lat(°)	Deep(km)	${{\varvec{M}}}_{{\varvec{r}}{\varvec{r}}}$(10^20^)	${{\varvec{M}}}_{{\varvec{\theta}}{\varvec{\theta}}}$(10^20^)	${{\varvec{M}}}_{\boldsymbol{\varphi }\boldsymbol{\varphi }}$(10^20^)	${{\varvec{M}}}_{{\varvec{r}}{\varvec{\theta}}}$(10^20^)	${{\varvec{M}}}_{{\varvec{r}}\boldsymbol{\varphi }}$(10^20^)	${{\varvec{M}}}_{{\varvec{\theta}}\boldsymbol{\varphi }}$(10^20^)
GCMT	85.33	27.91	12	1.76	− 1.82	0.0579	8.04	− 1.51	0.475
CPPT	84.66	28.26	12	0.98	− 1.09	0.11	5.05	− 1.12	0.24
USGS	84.731	28.231	10	1.63	− 1.622	0.019	6.339	− 0.944	0.429
GFZ	84.72	28.18	18	0.81	− 0.81	− 0.01	5.49	− 1.15	0.37

### Source parameters of the 2015 Mw7.8 earthquake in Nepal

The focal mechanism solution is determined based on the inversion of fault geometric parameters and average slip parameters by surface displacement, and the focal position is determined by the spectral element method with surface displacement as the constraint condition.

In the inversion of fault geometric parameters and average slip parameters, this study inverts the geometric parameters of coseismic faults based on the Okada model and Bayesian method(GBIS; http://comet.nerc.ac.uk/gbis/) with GNSS data constraints^26^. During the inversion, the reference coordinate origin is 84.731° E, 28.231° N. The range of geometric parameters for setting faults is as follows: the dip angle is 0° ~ 90°, the strike is 180° ~ 360°, the X coordinate of the midpoint of the lower edge of the fault is 40,000 ~ 150,000, and the Y coordinate is − 60,000 ~ 10,000. The fault length is 40 ~ 150 km, the fault width is 10 ~ 80 km, the depth is 2 ~ 60 km, and the number of iterations is 10^7^. After many iterations, the posterior probability density distribution of the fault geometric parameters is Gaussian. See Table 3 for the fault geometric parameters. In Table 3, the first column is the parameter name, the second, third, and fourth columns are the best quality, average, and median values of the parameters, and the fifth and sixth columns are the 2.5% and 97.5% confidence intervals.

**Table 3:** Geometric parameters of planar faults.

Parameter	Optimal	Mean	Median	2.5%	97.5%
Length (m)	100,652	100,639	100,638	99,951	101,341
Width (m)	38,207	38,192	38,192	38,087	38,295
Depth (m)	11,225	11,219	11,219	11,155	11,284
Dip (^∘^)	5.48	5.48	5.48	5.37	5.59
Strike (^∘^)	283.23	283.22	283.22	283.12	283.32
X (m)	69,953	69,942	69,942	69,665	70,214
Y (m)	− 18,551	− 18,557	− 18,557	− 18,659	− 18,454
Fault StrSlip (m)	− 0.03	− 0.03	− 0.03	− 0.04	− 0.02
Fault DipSlip (m)	5.19	5.19	5.19	5.18	5.20

**Figure 3:**
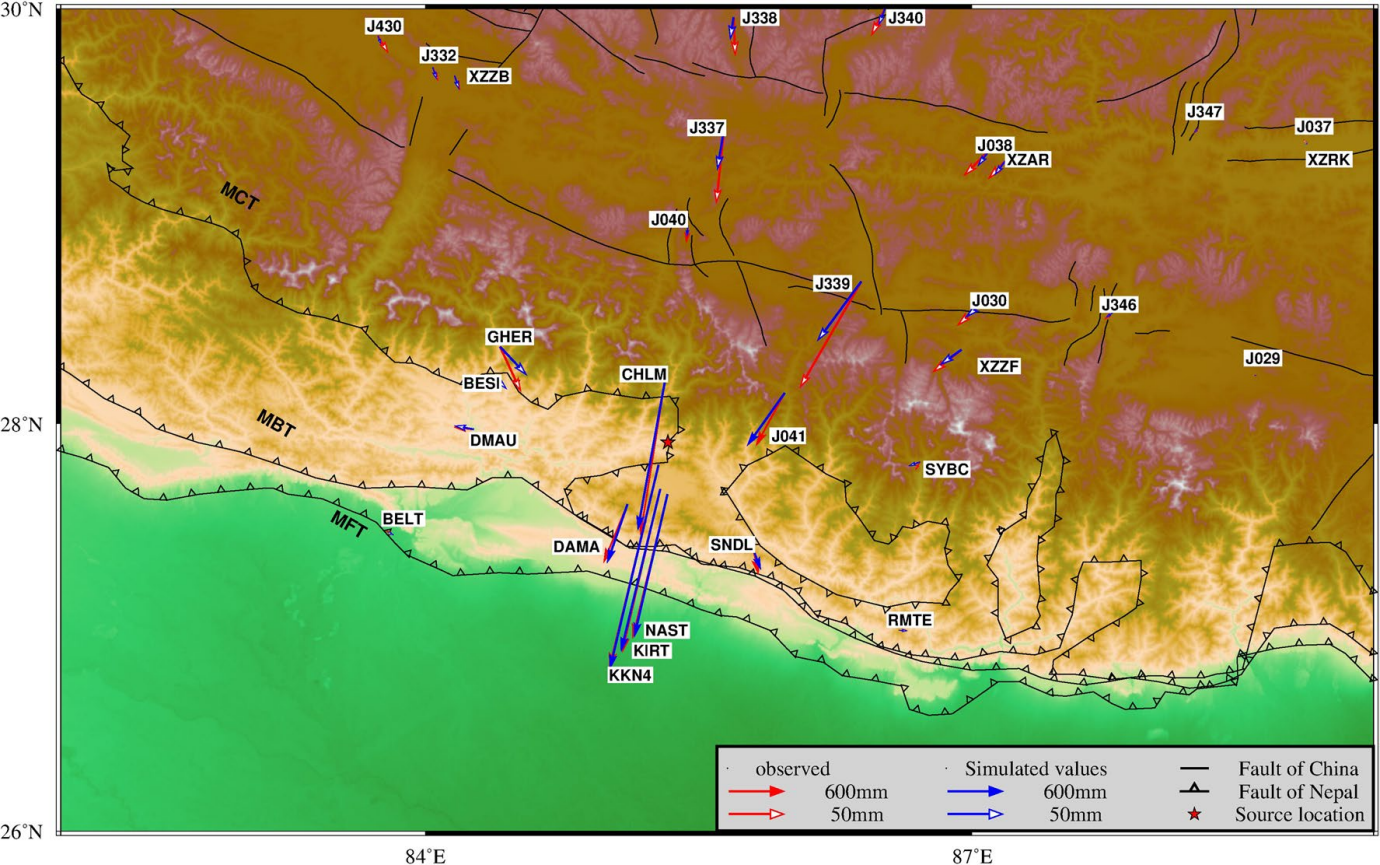
Comparison of coseismic GNSS displacement and model forward modeling results in Nepal. The red arrow in the figure is the horizontal displacement observed by GNSS, and the blue arrow is the horizontal displacement of the analog value. The length of the solid and hollow needles represents the displacement of 600 mm and 50 mm, respectively.

The fault parameters are consistent with most results^3,7,33,34^. We use the parameters in Table 3 to simulate the horizontal displacement of the surface, as shown in Fig. 3. The results agree with the GNSS observation results. In this paper, we regard GNSS observations as valid values and find that the fitting residuals of all stations in Nepal are within 4 cm, of which the maximum displacement station KKN4 has fitting residuals of 1.7 cm, and the average fitting residuals of stations in China are 3 cm. This shows that the inversion of fault geometric parameters is reliable.

**Figure 4:**
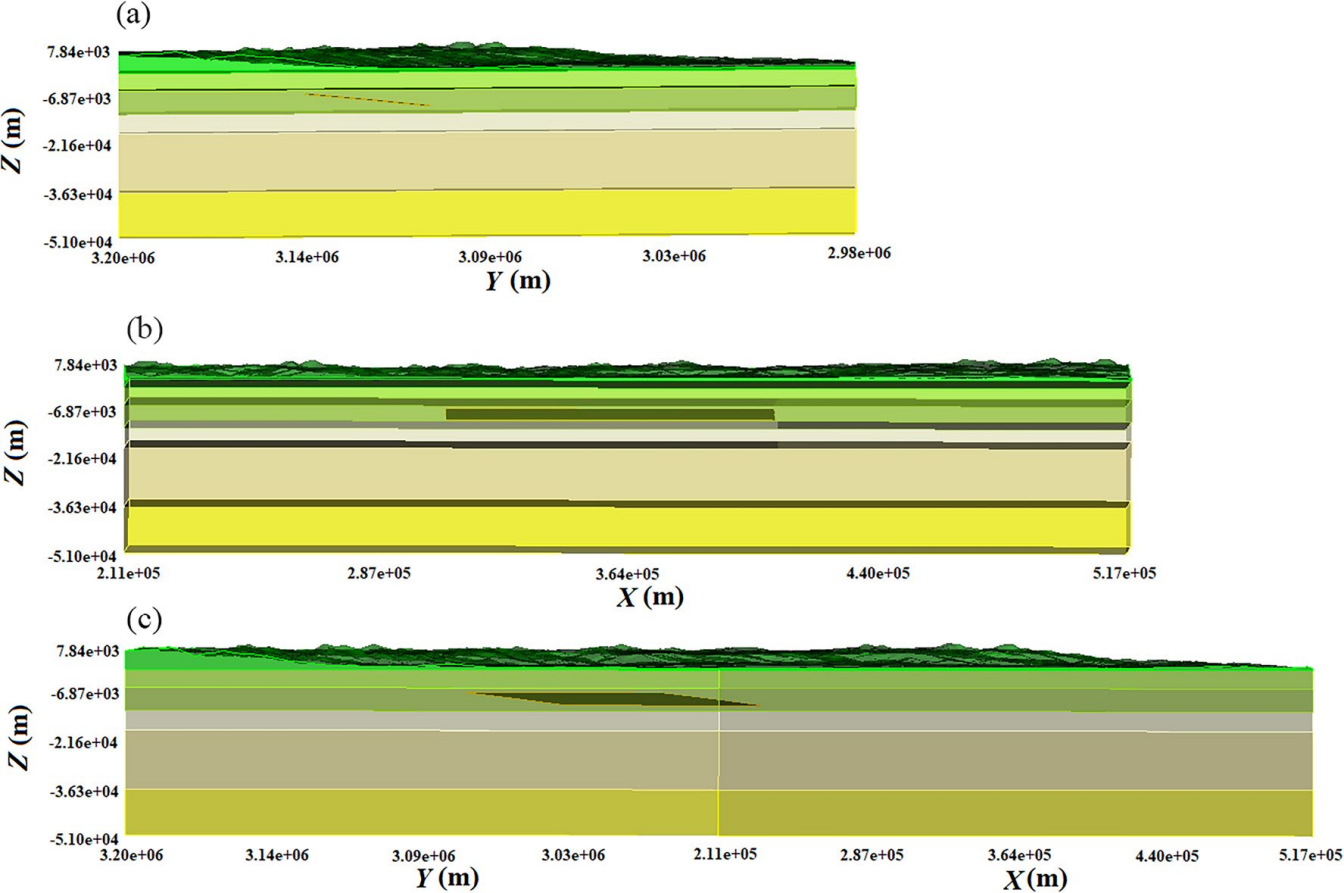
Three-dimensional models of Nepal. The figure is a three-dimensional model of Nepal. Different colors in the direction of the Z coordinate axis represent various media—Table 1 for its parameters.

First, in this paper, the extent of 290 km × 310 km × 50 km around the earthquake is taken as the study area and divided into 1,075,200 grid cells (excluding topography); we set artificial absorbing boundaries at the periphery and bottom of the grid^35^, and set the upper surface as a free boundary. In order to adapt to finer grid requirements, we added two doubling grid layers at depths of 6 km and 26 km; finally, a terrain grid containing 78,061 elevation points was added to complete the computational modeling (Fig. 4). The data were then processed for about 30 min using the SPECFEM-X program on a server with four parallel processors and 166 GB of operating memory.

**Figure 5:**
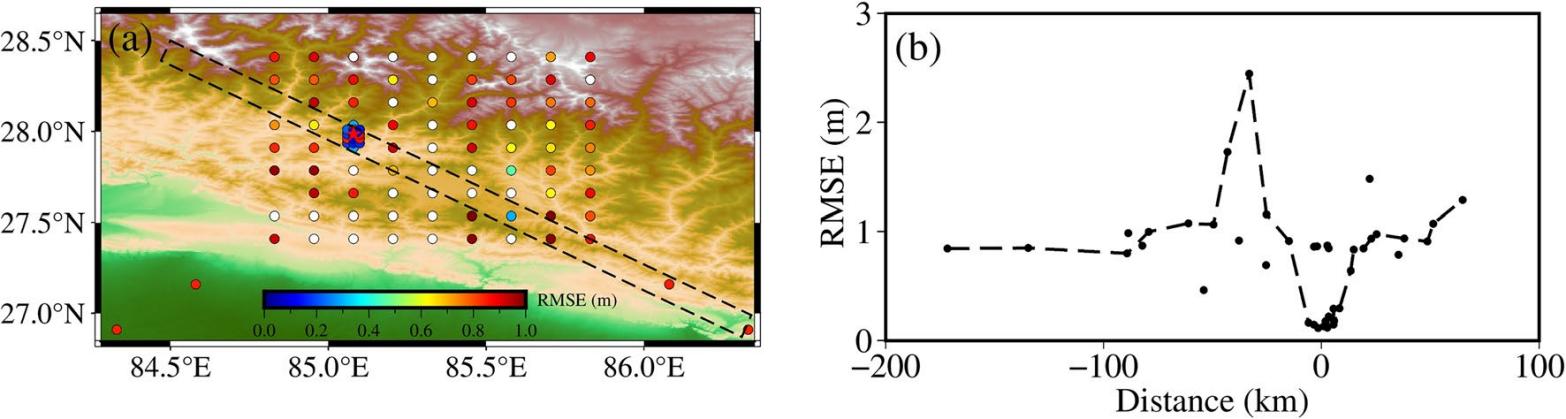
RMSE of forwarding coseismic displacement of source location. The dots in (**a**) are the searched source locations, the red pentagram is the optimal source location, and (**b**) shows the RMSE of the source locations in the black dashed box in (**a**). The horizontal axis in Fig. (**b**) represents the transverse distances, with (85.08°E, 27.985°N) as the origin, with the distance to the northeast being positive and the distance to the southwest being negative. The vertical axis represents the RMSE of the residuals of the simulated values.

When describing Focal Mechanism Solutions $r$, $\theta$, $\varphi$.To establish a spherical coordinate system, we use the geometric parameters in Table 3 to calculate the seismic moment ${M}_{0}=6.8e+20$ and moment tensor M(${M}_{rr}=1.2975e+20$,${M}_{\theta \theta }=-1.2298e+20$, ${M}_{\varphi \varphi }=-0.06766e+20$, ${M}_{r\theta }=6.4987e+20$, ${M}_{r\varphi }=-1.5242e+20$, ${M}_{\theta \varphi }=0.2885e+20$)^36^. Establish a 2° × 2° search region with the vertical displacement of GNSS stations close to the source (KKN4, KIRT, CHLM, NAST, DAMA) as the constraint, and the objective function is to minimize the residual of the simulated values. The final determined source position is (85.08° E, 27.985° N), shown in Fig. 5.

### Inversion of coseismic slip distribution

**Figure 6:**
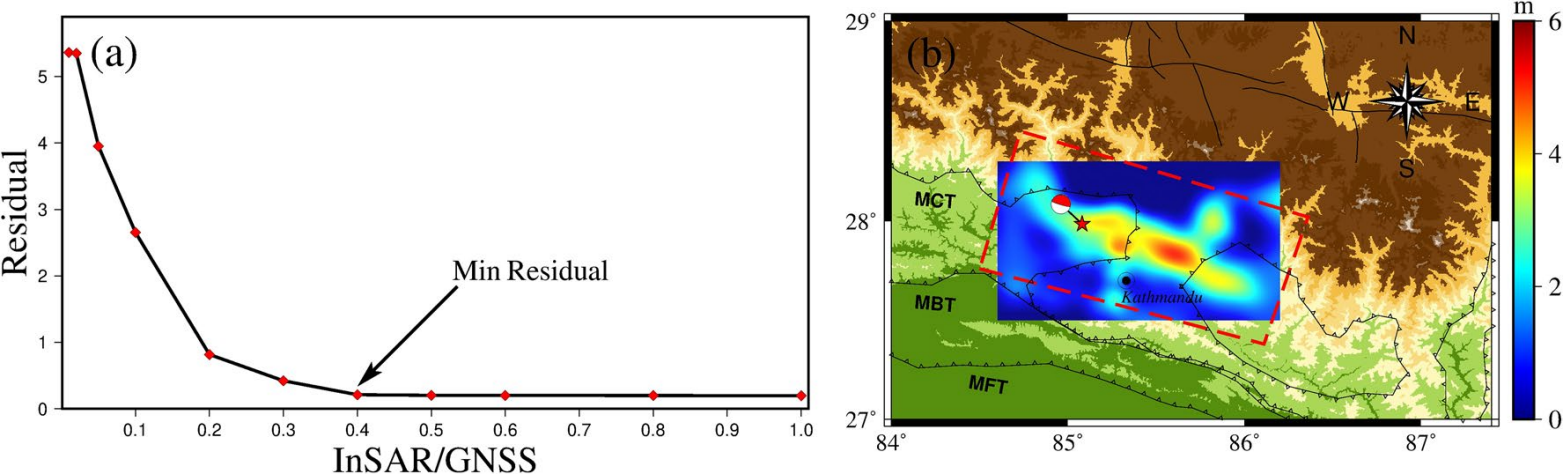
Fault slip displacement calculated by SDM. (**a**) shows the InSAR/GPS ratio used to determine the minimum error. (**b**) shows the red dashed box in the figure representing the fault location, and the middle plot shows the displacement of 1500 subfaults calculated by SDM.

In this paper, using the fault geometry inverted by the Bayesian algorithm as the input parameters and the GNSS and InSAR data as constraints, the slip distributions of the 1500 subfaults along the strike and dip directions, respectively, are computed using the SDM program^37^. At the same time, we constrain the amount of slippage between neighboring subfaults by applying Laplace smoothing. We determine the weight ratio of GPS to InSAR to be 1:0.4 by discounting the residuals of the GPS and InSAR fits^38^, as shown in Fig. 6. Calculate the moment tensor of each subfault using the following equation.

11 $$\begin{array}{*{20}c} {M_{0} = \mu DA} \\ \end{array}$$

12 $$\begin{array}{*{20}c} {M_{\theta \theta } = - M_{0} \left( {sin\delta cos\lambda sin2\phi + sin2\delta sin\lambda sin^{2} \phi } \right)} \\ \end{array}$$

13 $$\begin{array}{*{20}c} {M_{\theta \varphi } = - M_{0} \left( {sin\delta cos\lambda cos2\phi + 1/2sin2\delta sin\lambda sin2\phi } \right)} \\ \end{array}$$

14 $$\begin{array}{*{20}c} {M_{r\theta } = - M_{0} \left( {cos\delta cos\lambda cos\phi + cos2\delta sin\lambda sin\phi } \right)} \\ \end{array}$$

15 $$\begin{array}{*{20}c} {M_{\varphi \varphi } = + M_{0} \left( {sin\delta cos\lambda sin2\phi - sin2\delta sin\lambda cos^{2} \phi } \right)} \\ \end{array}$$

16 $$\begin{array}{*{20}c} {M_{r\varphi } = + M_{0} \left( {cos\delta cos\lambda sin\phi - cos2\delta sin\lambda cos\phi } \right)} \\ \end{array}$$

17 $$\begin{array}{*{20}c} {M_{rr} = + M_{0} sin2\delta sin\lambda } \\ \end{array}$$

where $\mu$ is the shear modulus, D is the fault displacement, and A is the fault area; $\delta$ denotes the fault dip (0° ≤ $\delta$ ≤ 90°), $\lambda$ denotes the slip angle of the fault (-180° ≤ $\lambda$ ≤ 180°), and $\phi$ represents the strike angle.

### Effect of topography on coseismic displacements

First, we establish three models: Model A represents the heterogeneous model with topography, Model B represents the heterogeneous model without topography, and Model C represents the homogeneity model without topography.

This paper uses the following formula to measure the impact of the terrain effect^13^:

18 $$\begin{array}{*{20}c} {P = \frac{{\left| {\Delta i} \right|}}{{\left| {j_{max} } \right|}}\cdot100\% } \\ \end{array}$$

where $\Delta i$ is the difference between the coseismic displacement of the calculation point considering the terrain factor and without considering the terrain factor, $j_{max}$ is the maximum value of coseismic displacement in all calculation points, and $P$ is the relative percentage of the topographic effect.

**Figure 7:**
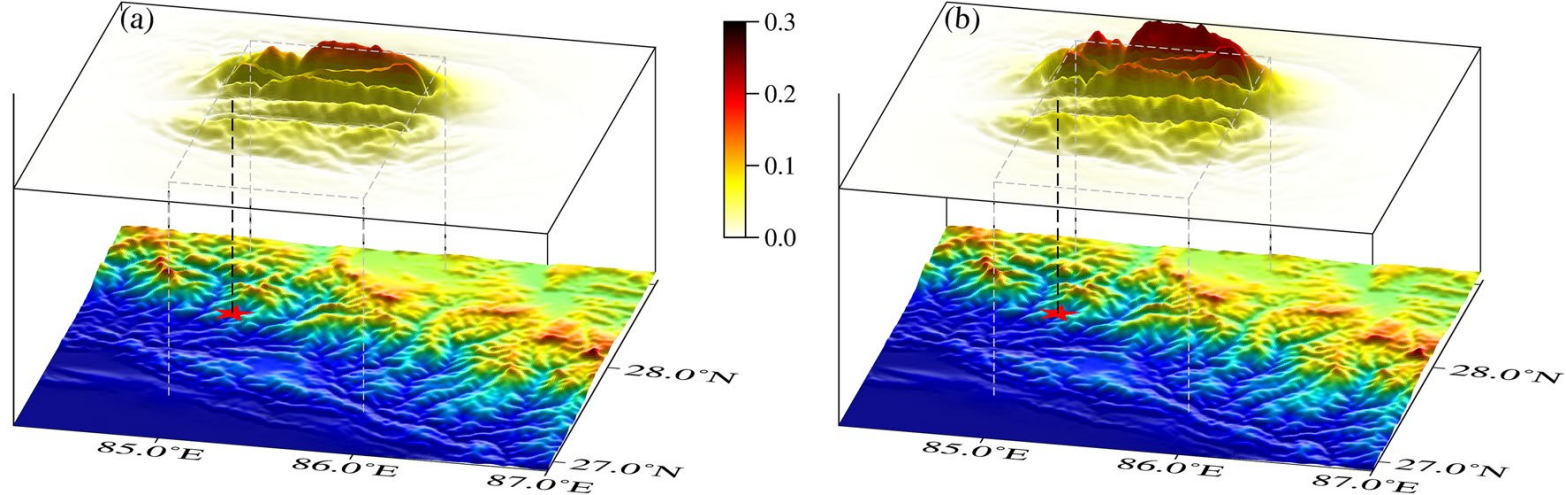
Influence of topography under a single-side source seismic source. (**a**) shows the difference between the two models under the influence of topographic factors for a single surface source, and (**b**) shows the value of the influence of the topographic factor.

**Figure 8:**
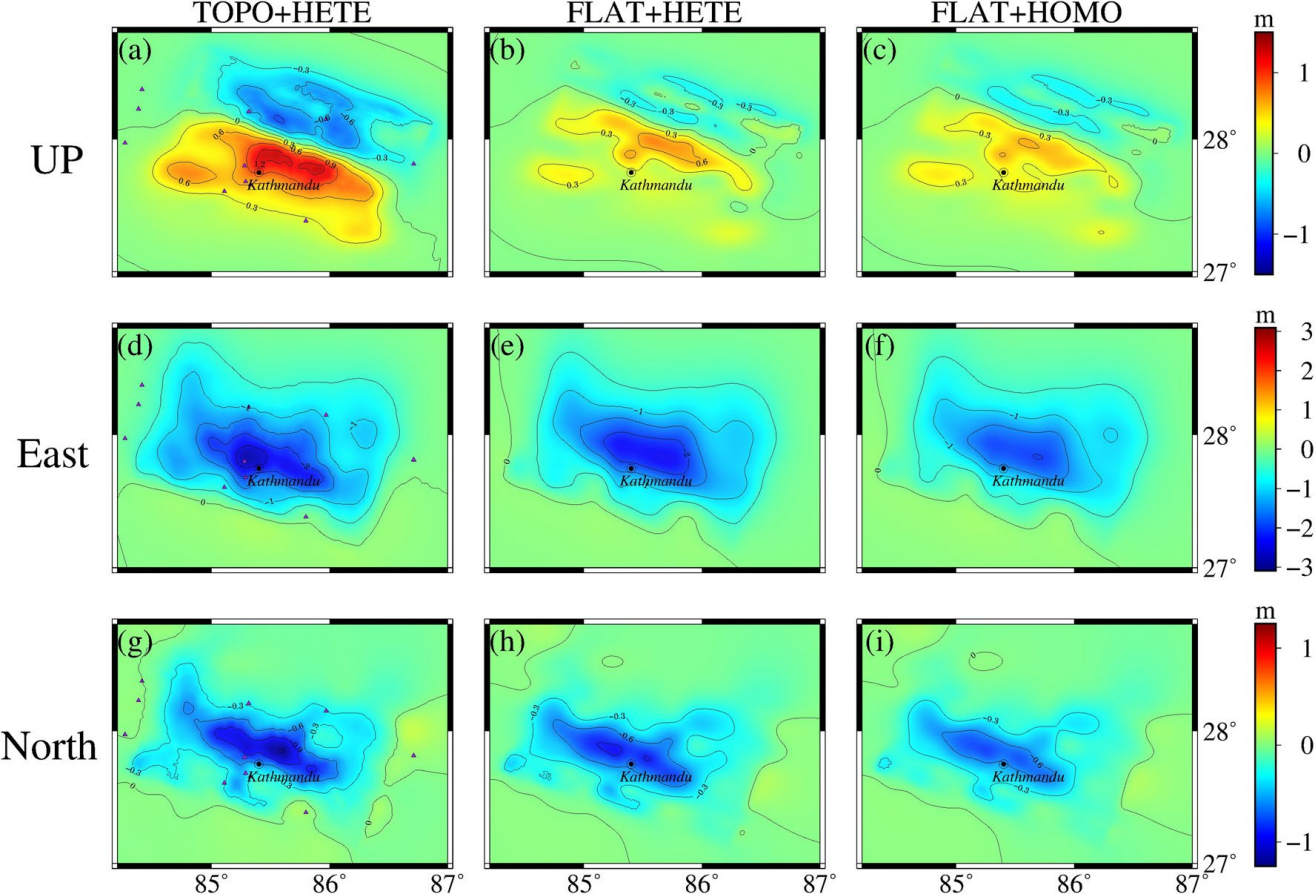
Surface coseismic displacements for different models under multiple subfault sources. (**a**–**i**) represents the three-component displacements based on three different models under multiple sub-fault sources and are positive in the north, east, and up directions. The purple triangles in (**a**,**d**,**g**) are GNSS stations within the Nepal.

In this section, the spectral element method calculates the coseismic surface displacements in the Nepal, considering topographic factors and without topographic factors, respectively. When using a single finite fault source, the influence of the topography is mainly reflected in the fault region in Nepal. It shows the morphology of the high north and the low south, which corresponds to the distribution of the topography in the region, and the effect of the topography produces a maximum of nearly 30%, as shown in Fig. 7. When multiple sub-faults are used as the source, the vertical displacement is analyzed in terms of the presence of two highlighted peaks in the direction of the fault motion, which corresponds to the backwash type of earthquakes. When using a topographic inhomogeneous model, the displacements are closer to the InSAR results processed by Lindesy^23^, as shown in Fig. 8a–c. Then, in the horizontal direction, the direction of the GNSS displacements is roughly the same as that of the GNSS displacements in Nepal. Moreover, by comparing the A and B models, it can be seen that there is a northward shift of the peak of the surface displacement of this earthquake, which also indicates that the presence of topography changes the position of the peak in the coseismic displacement of the surface of the forward earthquake. In summary, the influence of topography on the coseismic displacement is ~ 20%, which is larger than the influence of ~ 9% of topography based on the finite element method by Lin^11^, ~ 10% of topography based on the Bayesian method by Yang^7^, and ~ 6% of topography based on the InSAR data and finite element method by Wang and Fialko^39^. The effect of ~ 6% is all larger.

## Discussion

### PGV and PGA distribution under the influence of topographic factors

**Figure 9:**
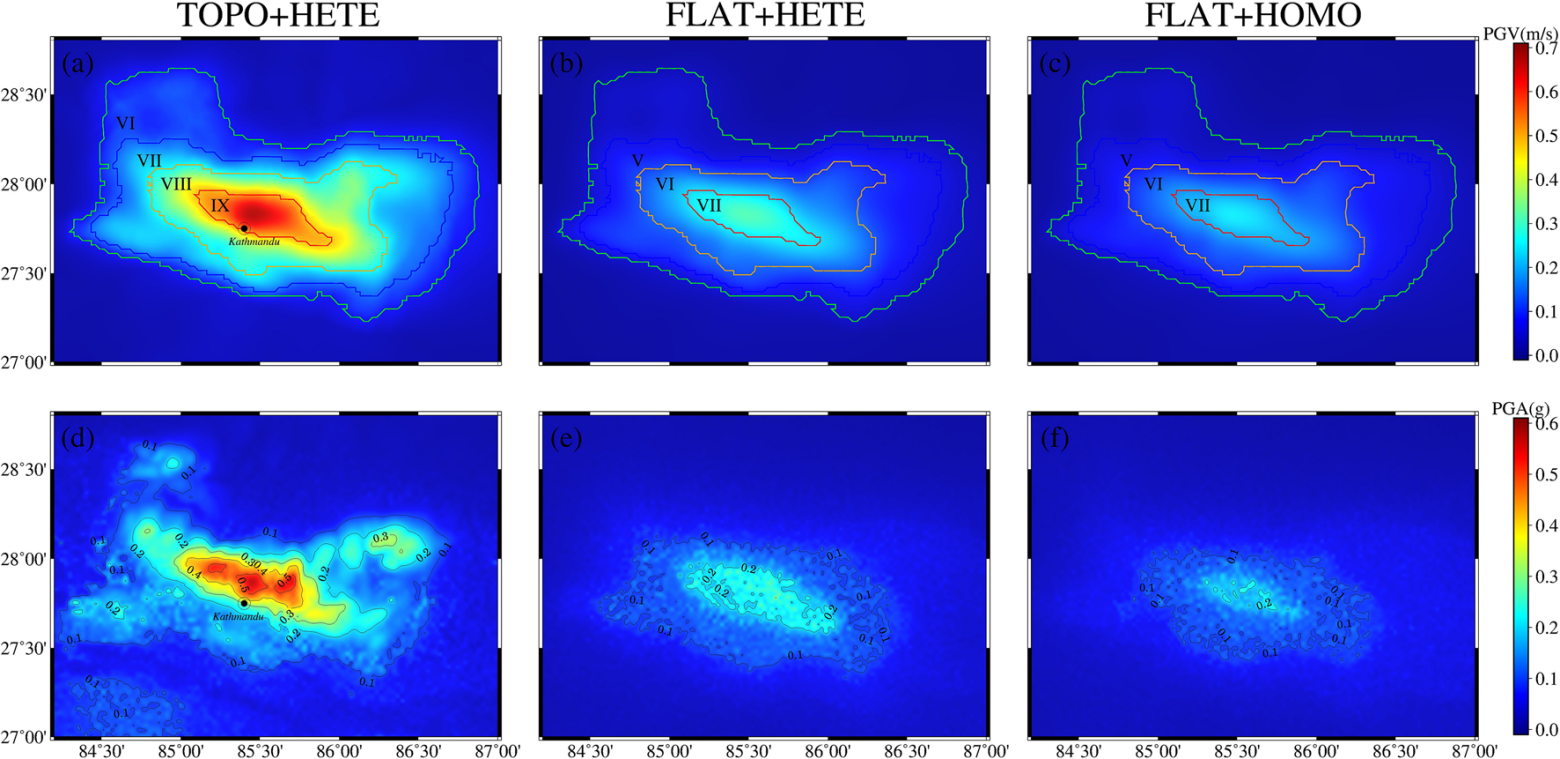
PGV and PGA distribution under the three model. (**a**–**f**) represent the PGV and PGA computed under three models. The areas with different colored contours in (**a**–**c**) represent the different intensity distributions, and the PGA in (**d**–**f**) is delineated by the 0.1 g contour.

Using the SPECFEM3D program developed by Komatitsch^10^, we calculate the PGV and PGA distribution in Nepal under the three models. From Fig. 9a, it can be seen that the area of this earthquake exceeding VI is more than 200,000 km^2^, which is consistent with the actual situation, and the capital city of Nepal is in the area of IX of intensity, and the hardest-hit areas with IX of intensity are all in the territory of Nepal; in Fig. 9b,c, the maximum intensity within Nepal under models B and C is about VII of intensity, which is far from IX and less than the PGV of model A. We see from Fig. 9d–f that the PGAs of Model A are all larger than those of Models B and C. We also compare with the published PGA distributions on the USGS(https://earthquake.usgs.gov/earthquakes/eventpage/us20002926/shakemap/pga) website, which we are in close agreement with the USGS published distribution of PGA > 0.5 g which is smaller. However all are concentrated near Kathmandu, and the distribution of PGA > 0.2 g. As for this earthquake, due to the low-angle retroflex type of earthquake, which is almost parallel to the ground, the intensity did not reach the imagined X or even XI.

### Effect of heterogeneous on coseismic earthquake

**Figure 10:**
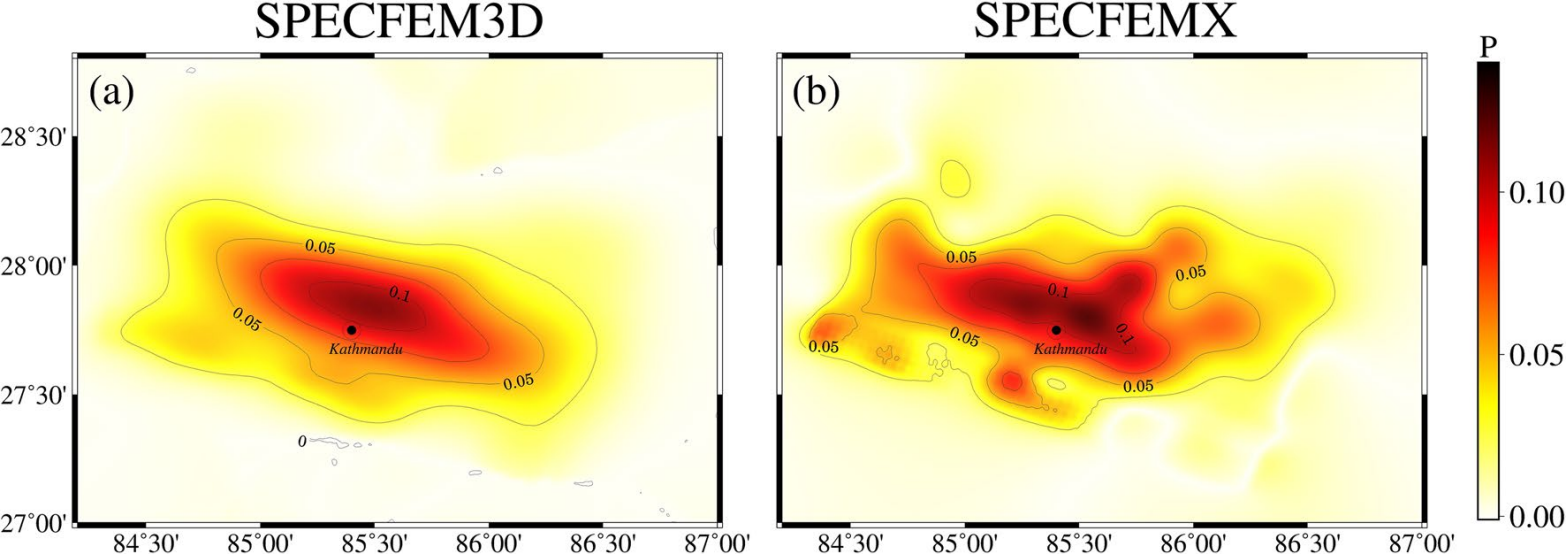
Influence of heterogeneous factors on orthogonal coseismic earthquakes. (**a**,**b**) show the influence of heterogeneous factors calculated using SPECFEM3D and SPECFEMX under the B and C models, respectively.

This section uses the control variable method to explore the effect of heterogeneous factors on forward coseismic earthquakes. Models B and C only change the longitudinal medium parameters, ensuring that the results show the effect of non-homogeneous factors rather than the overall differences in the models. Figures 8 and 9 show that the calculated PGV, PGA, and surface displacements in different media are more significant than those in a single medium. As can be seen in Fig. 10, the effects of non-homogeneous factors calculated using SPECFEM3D and SPECFEMX respectively are ~ 10% and less than the effects of topography in Nepal (the stress accumulation region between the location of the mainshock and the region of the largest aftershock).In contrast, Wang and Fialko considered the effects of non-homogeneous factors to be 10% and more than the effects of topography^39^, Sun considered the influence of radial non-homogeneous factors to be more than 25% ubiquitous^40^. This may be because the depth of the modeled area in this paper is too small relative to the previous two, and this earthquake is a shallow earthquake, which needs to adequately represent the effects of the deep Earth's media.

## Conclusions

Nepal is located in the southern foothills of the Tibetan Plateau. This area has significant terrain fluctuations and frequent seismic activity, providing good conditions for studying the impact of terrain factors on coseismic displacement. In previous studies of seismic activity, topographic factors are often ignored to simplify the model. This article uses GNSS data to constrain the Bayesian inversion algorithm that inverts fault geometry, activity parameters and the coseismic slip distribution using SDM. Then, we establish a three-dimensional elastic model to explore the influence of focal mechanism solution parameters and terrain factors on the coseismic displacement of the 2015 Mw7.8 earthquake in Nepal through the spectral element method. Based on the above, a new solution parameter for the earthquake source mechanism is given: the location of the epicenter is 85.08°E, 27.985°N, the depth of the epicenter is 11.2 km, the moment tensor is ${M}_{rr}=1.2975e+20$, ${M}_{\theta \theta }=-1.2298e+20$, ${M}_{\varphi \varphi }=-0.06766e+20$, ${M}_{r\theta }=6.4987e+20$, ${M}_{r\varphi }=-1.5242e+20$, ${M}_{\theta \varphi }=0.2885e+20$. Comparison of the results obtained from different models for the orthogonal modeling of this earthquake reveals that the effects of topographic and inhomogeneous factors are ~ 20% and ~ 10%, respectively, and that when using a point source, the effect on surface displacements tends to change drastically with a change in the location of the source, and it is also difficult to simulate the rupture mechanism of a large-scale earthquake; when using a single surface source as the source, the topographic factor is very significant and alters the location of the peak of the surface displacements; When topographic factors are not taken into account, surface displacements at the northern edge are underestimated, while displacements at the southern edge are overestimated. When using the heterogeneous terrain model, the simulated seismic intensity distribution range and surface displacements are close to reality. Although the results of this paper reflect the non-negligible influence of topographic factors on coseismic displacements, this paper does not consider lateral inhomogeneities, which will be taken into account in the following study in order better to investigate the influence of topographic factors on coseismic displacements.

### Supplementary Information

Supplementary Information 1.

Supplementary Information 2.

Supplementary Information 3.

## Data Availability

The datasets used and/or analysed during the current study are available from the corresponding author upon reasonable request.
